# Supplementary material for: Are carbon emissions trading and green financial instruments synergistic? -Comprehensive quantitative research based on content analysis
Source: PLoS One. 2024 Mar 7;19(3):e0298601. doi: 10.1371/journal.pone.0298601 (PMC10919601; doi:10.1371/journal.pone.0298601)
Supplement: S1 Data — (ZIP) [file pone.0298601.s001.zip › Data/地级市Z_F工作报告.zip!/10╘┬31╚╒╩2╛▌╗π╫▄.pdf]

公众号【马克数据网】数据汇总
10 月 31 日

一、会员附赠数据：

- 上亿条-海关产品进出口数据 2000-2021
- 工业企业数据库-处理 dofile+结果
- 工业企业数据库与海关数据匹配
- 工业企业数据库与专利数据匹配
- 工业企业数据库与污染数据匹配
- 全国省市县-企业新注册统计
- 全国省市县-有专利的企业统计（即将上线）
- 全国省市县-环保处罚案件统计
- 二手房成交数据-北京
- 二手房成交数据-天津
- 二手房成交数据-上海
- 二手房成交数据-重庆
- 《北京统计年鉴》整理为全市面板
- 《北京统计年鉴》整理为行业面板
- 《天津统计年鉴》整理为全市面板
- 《上海统计年鉴》整理为全市面板
- 《上海统计年鉴》整理为区县面板
- 《重庆统计年鉴》整理为全市面板
- 《重庆统计年鉴》整理为区县面板
- 上千万企业级-互联网招聘数据
- 国家统计局-全国年度面板
- 国家统计局-全国季度面板
- 国家统计局-全国月度面板
- 国家统计局-省级年度面板
- 国家统计局-省级季度面板
- 国家统计局-主要城市面板
- 《中国城市统计年鉴》整理为城市面板
- 《中国城市统计年鉴》整理为市辖区面板
- 《中国区域统计年鉴》整理为省份面板
- 《中国区域统计年鉴》整理为城市面板

- 《中国区域统计年鉴》整理为县域面板
- 《中国科技年鉴》整理为全国面板
- 《中国科技年鉴》整理为省份面板
- 《中国科技年鉴》整理为行业面板
- 《中国金融年鉴》整理为全国面板
- 《中国金融年鉴》整理为省份面板
- 《中国金融年鉴》整理为行业面板
- 《中国环境年鉴》整理为全国面板
- 《中国环境年鉴》整理为省份面板
- 《中国环境年鉴》整理为行业面板
- 北大法宝-环保行政处罚数据

更多高价值数据，陆续整理中

二、往期数据（会员无需转发）

地区数据

- 碳排放汇总-各国/各行业/各省份/各城市/各区县
- 全国-新能源汽车销量 2017-2021
- 全国各省份-117 个互联网和运输指标 2000-2019
- 全国各省份-94 个科技指标 2000-2019
- 全国各省份-139 个教育指标 2000-2019
- 全国各省份-13 个对外贸易指标 2000-2019
- 全国各省份-48 个能源指标数据 2000-2020
- 全国各省份-142 个创业、就业 1990-2019
- 全国各省份-结婚和离婚数据 2000-2019
- 全国各省份-企业法人单位数 2010-2019
- 全国各省份-计算平均受教育年限 1987-2019
- 全国各省份-执业律师人数 2000-2018
- 全国各省份-PPP 项目数据 2016-2021
- 全国各省份-科技年鉴整理 2005-2019
- 全国各省份-环境年鉴整理 2005-2018
- 地级市-绿色 TFP 原始数据 2003-2018
- 地级市-创新创业指数 1990-2019
- 地级市-高铁开通数据 2001-2019
- 地级市-固定资产投资 1990-2018

- 地级市-污染排放指标 1990-2019
- 地级市-环境指标数据 2007-2017
- 地级市-商品房销售价格 2010-2020
- 地级市-进出口总额数据 2003-2018
- 地级市-地方财政收支数据 1990-2019
- 地级市-公路里程数据 1978-2020
- 地级市-PM2.5 数据 2000-2018
- 地级市-汽车拥有量 2001-2020
- 区县-财政收支数据 2000-2019
- 区县-PM2.5 数据 2000-2018
- 区县-CO2 排放 1997-2017
- 区县-房价数据 2011-2020
- 地级市-三种空间权重矩阵及其标准化
- 全国各省-资源和环境 181+指标 2003-2019
- 全国各省-贪污腐败数据 1998-2016
- 区县-人口、户数及城镇化率 2000-2019
- 地级市-城镇化率 2000-2020 年
- 全国/省/市/县-互联网普及率及用户 1997-2020
- 地级市-财政收支与透明度 2003-2020
- 各省/地级市-政府工作报告文本 2003-2020
- 省/市/县-数字普惠金融指数/数字经济指数/绿色金融指数/县域数字乡村指数 2011-2020
- 地级市-用水、用电、用气数据 2001-2020
- 省/市/县-固定资产投资数据~2020
- 人口抽样调查样本数据 1990-2019
- 各省/市/县经纬度、区县与地级市和省会的距离
- 各省市政府工作报告、环境词汇词频分析
- 省级/地级市增长目标 2000-2019
- 各省/城市-空间相邻数据
- 国家知识产权局-地级市绿色专利申请量 2005-2019
- 各省份-财政收支分类明细数据 2000-2020
- 各地级市/区县-平均工资数据 1996-2019
- 全国各省份-人口抽样调查样本数据 2002-2019
- 地级市-逆温数据 2000-2021

- 各省/地级市-空间距离及公路交通距离数据
- 各省/地级市-收入差距泰尔指数与公式 2000-2019

企业数据

- 上市公司全要素生产率-原始数据+代码+结果 2000-2019
- 上市公司-绿色专利申请 1985-2019
- 上市公司-绿色专利授权 1985-2019
- 上市公司-研发投入与专利数据 2007-2020
- 上市公司排污费数据 2010-2019
- 上市公司环保支出数据 2010-2019
- 上市公司财务报表数据 1991-2019
- 上市公司数据及高管信息数据 1990-2020
- 上市公司社会责任评分数据 2010-2020
- 工业企业数据 1998-2014
- 工业企业与国家知识产权局专利匹配
- 海关进出口数据 2000-2015
- 5W 家创业公司和投资的信息 2006-2015
- 上市公司政府补助数据 2003-2019
- 上市公司对外投资数据 2007-2021
- 上市公司环境排放明细数据 2008-2020
- 上市公司高管出生地、教育和薪酬 1990-2021
- 工业企业数据 1998-2015
- 环境信息披露指数 EDI2015-2019
- 海关与上市公司匹配数据 2000-2016
- 上市公司专利申请&授权、绿色专利申请&授权、专利引用 1985-2020
- 企业境外投资名录 1983-2015
- 上市公司年报、各省/地级市政府报告文本
- 上市公司能源消耗数据 2008-2020
- 上市公司总经理董事长政治关联数据 2008-2020
- 上市公司-环境投资明细数据 2009-2020
- 2200+上市公司仲裁数据 2000-2021
- 上市公司-知识产权数据 2013-2019
- 上市公司-专利号、申请、授权明细 2007-2020
- 上市公司 1100+变量数据处理大全 1990-2019
- 彭博 ESG 综合得分、分项得分 2011-2020

银行数据

- 3300+银行数据大全 1954-2020
- 商业银行网点数据 1949-2019
- 全国各省份-银行网点数量 2005-2019
- 全国各省份-金融网点和从业人数 2007-2018
- 全国各区县-金融网点统计数据~2020
- 800+商业银行财务面板数据 2008-2020

爬虫数据

- 百度迁徙大数据整理版 2020+2019 同期
- 链家-住房交易微观数据 2002-2018
- 人人贷-真实贷款数据 2013-2015
- 全国工商企业信息注册数据 589 万条
- 232 万条土地交易微观数据 2000-2020
- 百度指数-每日数据 2011-2021
- 每日新闻联播-文字整理版 2015-2021
- 全国 18 个城市地理 POI 数据
- 141 万+招聘爬虫数据-北京
- 133 万+招聘爬取数据-上海
- 北京市各区县-二手房均价 2011-2020
- 全国 3445 个地区-地理位置数据
- 全国 11970 条-A 级景区数据
- 地级市-空气质量数据 2013-2021
- 10W 条知乎评论数据
- 各省市-人民网领导留言版数据（至 2020.12）

测算数据

- 各省全要素生产率-原始数据+测算+结果 2006-2019
- 樊纲市场化指数-原始数据+测算 1997-2019
- 各省城镇与农村恩格尔系数 1978-2019
- 322 个地级市-恩格尔系数 1978-2019
- 地级市资本存量-永续盘存+测算 2002-2017
- 工具变量：杭州到各城市的球面距离(含测算)
- 上市公司治理水平-数据+代码+结果 2003-2019
- 上市公司融资约束-SA 测算+结果 2000-2019
- 高管团队异质性-原始+代码+结果 2008-2019

- 管理者能力测算数据+代码 2010-2019
- 环境不确定性数据和代码 2000-2020
- 空间计量模型：SDM、SEM、SAR 模型、LM、Wald、LR 检验、Moran's 计算
- 三废排放量和熵值法计算环境规制综合指数 1997-2019
- 用 Stata 计算 Moran's I 莫兰指数
- 合成控制法 SCM 案例含安慰剂检验
- PSM-DID 方法 stata 操作详解
- 工业企业全要素生产率 TFP 估计
- 全要素生产率-上市公司/中国/各省/各地级市 1949-2020
- 空间杜宾模型-相关检验及结果分析
- DEA 模型大全-含四类 DEA 模型的视频教程和案例演示、do 文件、数据
- 面板门槛回归模型数据与代码 2012-2016
- 【文献+代码】多期 DID 和事件研究法

其他数据

- 省市县-官员任期/官员腐败/县级干部数据 1945-2020
- 地市级市委书记及市长数据库 2000-2012
- 中国经济政策不确定性指数 2000-2021
- 一带一路国家-对外投资 1970-2019
- 世界银行 WDI 数据库 1960-2020
- 各行业-赫芬达尔指数表 1990-2020
- 中国企业层面微观 OFDI2005-2020
- 中国企业调查数据 2005&2012
- 中国企业海外并购数据 2002-2019
- 中国商务部境外投资企业名录 1980-2013
- 人民日报-新闻语料库 1946-2003
- 各高校各专业-考研历年分数线 2017-2020
- 第一至七次全国人口普查资料 1953-2020
- 世界、全国、各省-投入产出表 1990-2018
- 工业企业的专利原始数据 1998-2013
- 企业专利被引用数据 2000-2020
- Stata17 最高配 MP 版本-win&mac
- 碳中和数据全集！碳排放、碳封存、碳排放权配额、碳交易、能源消费等 8 份数据

- 【文章+数据+程序】中国工业经济论文合集 2015-2021
- 经济学顶刊 JPE 论文合集 2011-2019

扫码关注公众号【马克数据网】
